# The Biphasic Increase of PIP2 in the Fertilized Eggs of Starfish: New Roles in Actin Polymerization and Ca^2+^ Signaling

**DOI:** 10.1371/journal.pone.0014100

**Published:** 2010-11-23

**Authors:** Jong T. Chun, Agostina Puppo, Filip Vasilev, Giovanni Gragnaniello, Ezio Garante, Luigia Santella

**Affiliations:** Stazione Zoologica Anton Dohrn, Villa Comunale, Napoli, Italy; CNRS UMR6543, Université de Nice, France

## Abstract

**Background:**

Fertilization of echinoderm eggs is accompanied by dynamic changes of the actin cytoskeleton and by a drastic increase of cytosolic Ca^2+^. Since the plasma membrane-enriched phospholipid phosphatidylinositol 4,5-bisphosphate (PIP2) serves as the precursor of inositol 1,4,5 trisphosphate (InsP_3_) and also regulates actin-binding proteins, PIP2 might be involved in these two processes.

**Methodology/Principal Findings:**

In this report, we have studied the roles of PIP2 at fertilization of starfish eggs by using fluorescently tagged pleckstrin homology (PH) domain of PLC-δ1, which has specific binding affinity to PIP2, in combination with Ca^2+^ and F-actin imaging techniques and transmission electron microscopy. During fertilization, PIP2 increased at the plasma membrane in two phases rather than continually decreasing. The first increase was quickly followed by a decrease about 40 seconds after sperm-egg contact. However, these changes took place only after the Ca^2+^ wave had already initiated and propagated. The fertilized eggs then displayed a prolonged increase of PIP2 that was accompanied by the appearance of numerous spikes in the perivitelline space during the elevation of the fertilization envelope (FE). These spikes, protruding from the plasma membrane, were filled with microfilaments. Sequestration of PIP2 by RFP-PH at higher doses resulted in changes of subplasmalemmal actin networks which significantly delayed the intracellular Ca^2+^ signaling, impaired elevation of FE, and increased occurrences of polyspermic fertilization.

**Conclusions/Significance:**

Our results suggest that PIP2 plays comprehensive roles in shaping Ca^2+^ waves and guiding structural and functional changes required for successful fertilization. We propose that the PIP2 increase and the subsequent formation of actin spikes not only provide the mechanical supports for the elevating FE, but also accommodate increased membrane surfaces during cortical granule exocytosis.

## Introduction

Starfish oocytes arrested at the first prophase of meiosis are characterized by a large nucleus (germinal vesicle, GV). When exposed to the maturation hormone (1-methyladenine, 1-MA), the oocytes reenter the cell cycle and proceed with meiosis to become mature eggs. The eggs of starfish and nearly all animal species display intense mobilization of intracellular Ca^2+^ at fertilization [1]. Being large and transparent, starfish eggs are also adequate to monitor other cytological changes occurring at fertilization [2].

The massive Ca^2+^ release in fertilized eggs in part facilitates exocytosis of cortical granules. The initial rise of Ca^2+^ induced by the sperm occurs at the egg cortex (cortical flash), and is followed by the propagation of Ca^2+^ waves starting from the site of sperm interaction [3], [4]. The release of Ca^2+^ from internal stores is mediated by several second messengers, i.e., InsP_3_, cyclic ADP-ribose (cADPr), and nicotinic acid adenine dinucleotide phosphate (NAADP), which bind to the cognate cytoplasmic receptors functioning as ligand-gated Ca^2+^ channels [5]–[7]. In starfish eggs, NAADP and InsP_3_ may play distinct roles in priming (NAADP) and propagating (InsP_3_) the Ca^2+^ signals [3], [8]. It is generally believed that the exocytosed contents of the cortical granules deposited in the perivitelline space contribute to formation of the fertilization envelope that serves as a mechanical barrier to block polyspermy [9].

In recent studies, however, it has been shown that fine regulation of the subplasmalemmal actin cytoskeleton is also required for exocytosis in neuroendocrine cells and fertilized eggs, as well as in non-excitable cells [10]–[14]. Ca^2+^ plays a role in remodeling the actin cytoskeleton through the actin-binding proteins whose activity is regulated by Ca^2+^, e.g. gelsolin, but conversely the actin cytoskeleton itself may modulate the efficacy of the intracellular Ca^2+^-releasing mechanisms [15]–[17]. With starfish eggs, we have demonstrated actin-dependent modulation of intracellular Ca^2+^ signaling in several different experimental paradigms [18]–[20]. In particular, the actin-binding protein cofilin substantially augmented intracellular Ca^2+^ release at fertilization while abolishing the cortical flash [20]. Hence, the fine regulation of the actin networks in the specific subcellular sites is likely to play pivotal roles both in Ca^2+^ signaling and in exocytosis [13], [14].

A growing body of evidence has suggested that PIP2, a phospholipid enriched at the plasma membrane, serves not only as a metabolic precursor of InsP_3_ [21], but also as a signaling molecule mediating diverse cell functions such as actin polymerization, regulation of ion channels, assembly and disassembly of vesicular coats, and mRNA processing [22]–[26]. By use of its negatively charged inositol head group, PIP2 recruits various proteins to the plasma membrane [27]. Interacting with actin-binding proteins, PIP2 regulates polymerization-depolymerization dynamics of microfilaments [28]. Hence, local concentrations of PIP2 may be used as a determinant for modulating the actin cytoskeleton and its related functions. However, the additional roles for PIP2 at fertilization, other than serving as a substrate for phospholipase C, are not well known. Despite its expected decrease [29], earlier studies had documented a significant increase of PIP2 in the fertilized eggs of sea urchin [30] and mouse [31]. Surprisingly, the surge of InsP_3_ at fertilization in echinoderm eggs has been found to take place much after the peak of Ca^2+^ release [29], [32]. In this regard, clear establishment of the spatiotemporal relationship between Ca^2+^ signaling and the local PIP2 levels would provide invaluable first insights.

The PH domain of PLC-δ1 binds to PIP2 with high affinity and specificity, and has thus been widely used as a marker to visualize this phospholipid [33]–[37]. The specific binding of the PH domain to PIP2 should, in principle, decrease the functionality of the accessible PIP2 at the plasma membrane. Hence, the PH domain of PLC-δ1 has also served as a specific molecular tool to sequester PIP2 [38]–[39]. In this contribution, we have investigated the roles of PIP2 at fertilization of starfish eggs by using the PH domain of PLC-δ1 as a PIP2-sequestering marker. At lower doses, the PH fusion protein successfully traced the spatiotemporal changes of PIP2 at fertilization and indicated that PIP2 levels indeed have a dual response at fertilization: a brief early rise and fall followed by a prolonged increase at the egg surface. The late increase was closely linked to the formation of numerous microfilament spikes that may help thrust the elevating vitelline layers during cortical granule exocytosis. At the higher dose that saturated PIP2 binding at the plasma membrane, the PH fusion protein also had an effect on the structure of the subplasmalemmal actin cytoskeleton, and significantly altered the dynamics of Ca^2+^ signaling inside the egg.

## Results

### PH-GFP and RFP-PH specifically bind to plasma membrane PIP2 in starfish eggs

**Figure 1:**
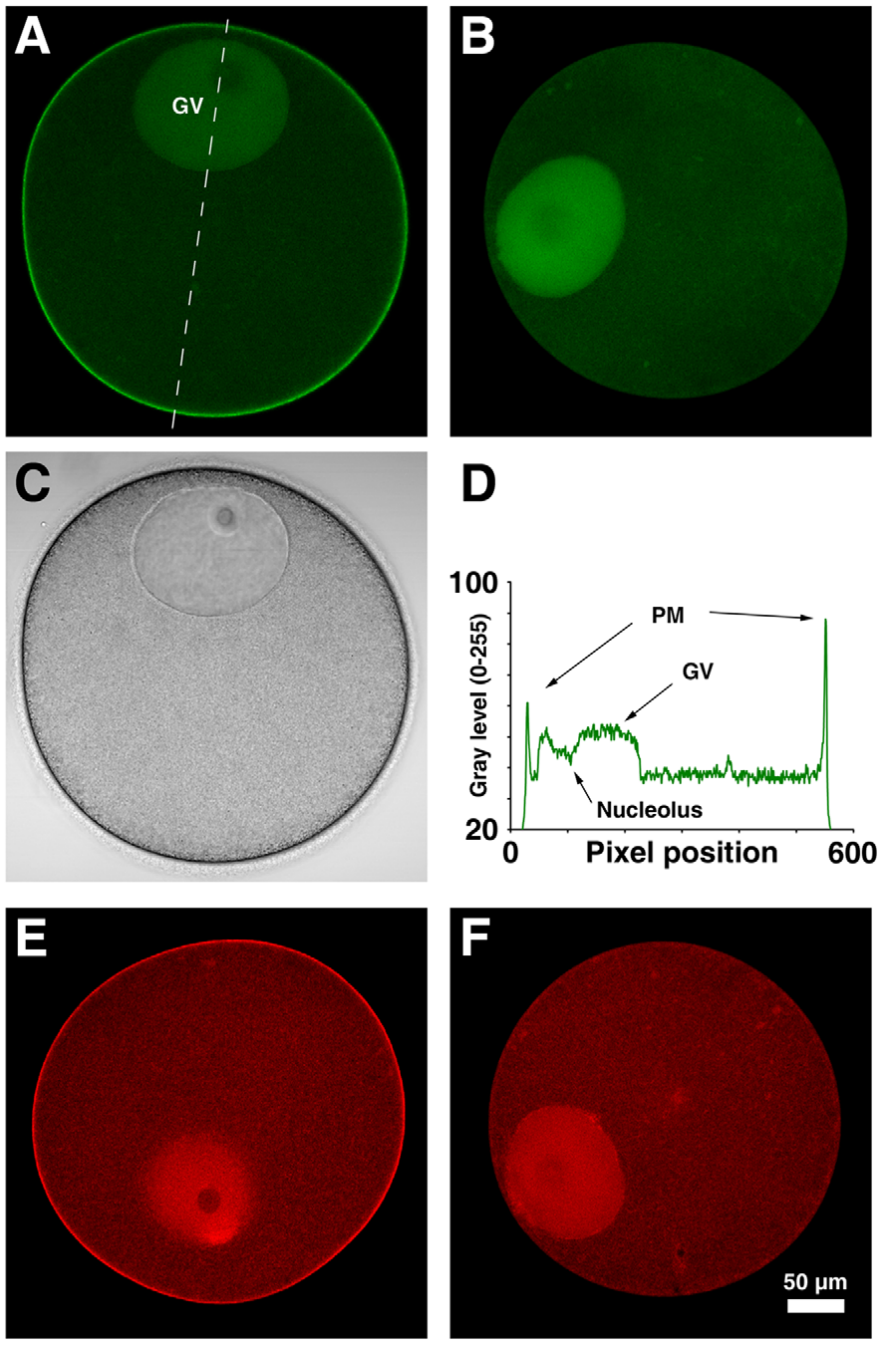
The PH domain of PLC-δ1 specifically bind to plasma membrane PI(4,5)P2 in starfish oocytes. Immature oocytes of *A. aranciacus* were microinjected with PH-GFP (**A**) or RFP-PH (**E**) fusion proteins (150 µM, pipette concentration), and the equatorial plane was monitored with confocal microscopy. The control proteins (R40A mutants) without the capability of PIP2-binding were not localized to the plasma membrane (**B**, **F**). (**C**) Bright field view of the same oocyte microinjected with RFP-PH in panel A. (**D**) The line intensity profile of the PH-GFP signals corresponding to the interrupted line in panel A. Abbreviation: PM, plasma membrane; GV, germinal vesicle. Scale bar, 50 µm.

To visualize PIP2 in the presence of other fluorescent probes for cytosolic Ca^2+^ (Calcium Green) and F-actin (Alexa Fluor 488-phalloidin), the PH domain of PLC-δ1 was tagged with either RFP or GFP. Within 5–10 minutes after microinjection into *Astropecten aranciacus* oocytes, both PH-GFP and RFP-PH were predominantly localized at the plasma membrane (Fig. 1A,E). The preferential distribution most likely represents their specific binding to PIP2, as the mutant probes (R40A) unable to bind PIP2 [29] failed to yield signals at the plasma membrane (Fig. 1B,F). Curiously, PH-GFP and RFP-PH also accumulated in the germinal vesicles (GV) (Fig. 1A,D,E), as was observed in the nucleus of mammalian cells [37]. However, whether these signals represent specific binding to PIP2 was questionable because the R40A probes also displayed similar patterns of distribution in the GV (Fig. 1B,F). Taken together, these results assure that both PH-GFP and RFP-PH probes specifically bind PIP2 at the plasma membrane of starfish oocytes whether the fluorescent tag was fused to the N-terminal (RFP-PH) or C-terminal (PH-GFP) side of the PH domain.

### The sperm-induced intracellular Ca^2+^ release is accompanied by the changes of PIP2 at the plasma membrane

**Figure 2:**
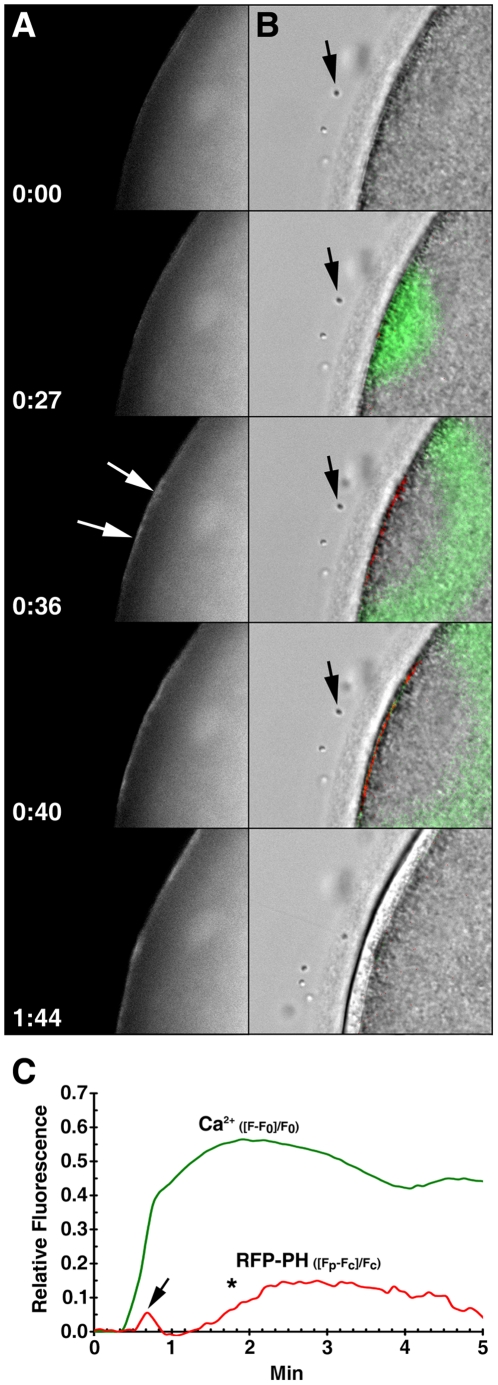
During fertilization, the intracellular Ca^2+^ release is followed by the biphasic increase of PIP2 at the plasma membrane. Mature eggs loaded with Calcium Green were microinjected with RFP-PH (150 µM, pipette concentration) to monitor the changes of PIP2 and sperm-induced Ca^2+^ signals. A representative result from 15 independent experiments was presented. (A) Changes of RFP-PH signals at the site of fertilization. The moment the spermatozoon is stopped at the egg surface was set to t = 0:00 (min:sec). The onset of PIP2 increment is evident by 0:36 (arrows). (B) Images of instantaneous increment (F*_inst_* = [(F_t_-F_t−1_)/F_t−1_]) in Ca^2+^ release (green) and PIP2 (red) were merged with the light transmission photomicrographs of the fertilized egg at the corresponding time points. The position of the fertilizing sperm was indicated with an arrow. (C) Temporal relationship between sperm-induced Ca^2+^ signaling (F_rel_ = [F−F_0_]/F_0_; green curve) and the fluctuation of the relative fluorescence (F_rel_ = [F_pm_−F_ct_]/F_ct_; red curve) for plasma membrane PIP2 in the region of the framed area of the sperm-egg interaction.

Mature eggs of *A. aranciacus* were loaded with RFP-PH and Calcium Green and inseminated to monitor the changes in PIP2 and cytosolic Ca^2+^ levels at the time of fertilization. By 36 sec after the sperm-egg contact, the labeling of PIP2 at the egg plasma membrane was markedly increased near the site of sperm interaction (Fig. 2A, white arrows). At this time, the sperm head was still located outside the jelly coat (Fig. 2B, arrow). When the data were analyzed for the incremental changes of the Ca^2+^ and PIP2 levels to focalize the sites of instantaneous rises by applying the formula (F*_inst_* = [(F_t_-F_t−1_)/F_t−1_]), it was evident that the initial increase of PIP2 took place at the sperm interaction site 9 sec after the sperm-induced Ca^2+^ release (see the images at 0:27 and 0:36 in Fig. 2B). When evaluated in reference to the level in the cytoplasm, the plasma membrane PIP2 levels displayed a dual response to the fertilizing sperm: the quick initial rise and fall and the prolonged late increase (Fig. 2C). The relative level of PIP2 at the plasma membrane (F_rel_  = [F_p_-F_c_]/F_c_) increased after fertilization, but it took place nearly 10 sec after the onset to the Ca^2+^ signaling (Fig. 2C, arrow). After the sharp increase of Ca^2+^ over a certain level (>0.3 R.F. unit), the relative level of PIP2 at the plasma membrane (F_rel_ values) began to decrease at 40 sec (Fig. 2C, arrow) and continued to subside to the negative numbers until about 60 sec, implying that much of the membrane-bound RFP-PH was translocated to the cytoplasm. This period of steep decrease of the RFP-PH signals at the plasma membrane corresponds to 80–100 sec after the addition of sperm, and the result is in general agreement with the timing of the accelerated InsP_3_ production observed in the fertilized eggs of the sea urchin species such as *A. crassispina*, *H. pulcherrimus*, and *L. pictus* [29], [32]. Subsequently, however, the F_rel_ values of plasma membrane PIP2 of the starfish egg gradually increased again (Fig. 2C, asterisk) and remained elevated for five min after fertilization.

### The sustained increase of PIP2 at the plasma membrane of fertilized eggs is linked to the formation of actin-filled spikes and the fertilization envelope

**Figure 3:**
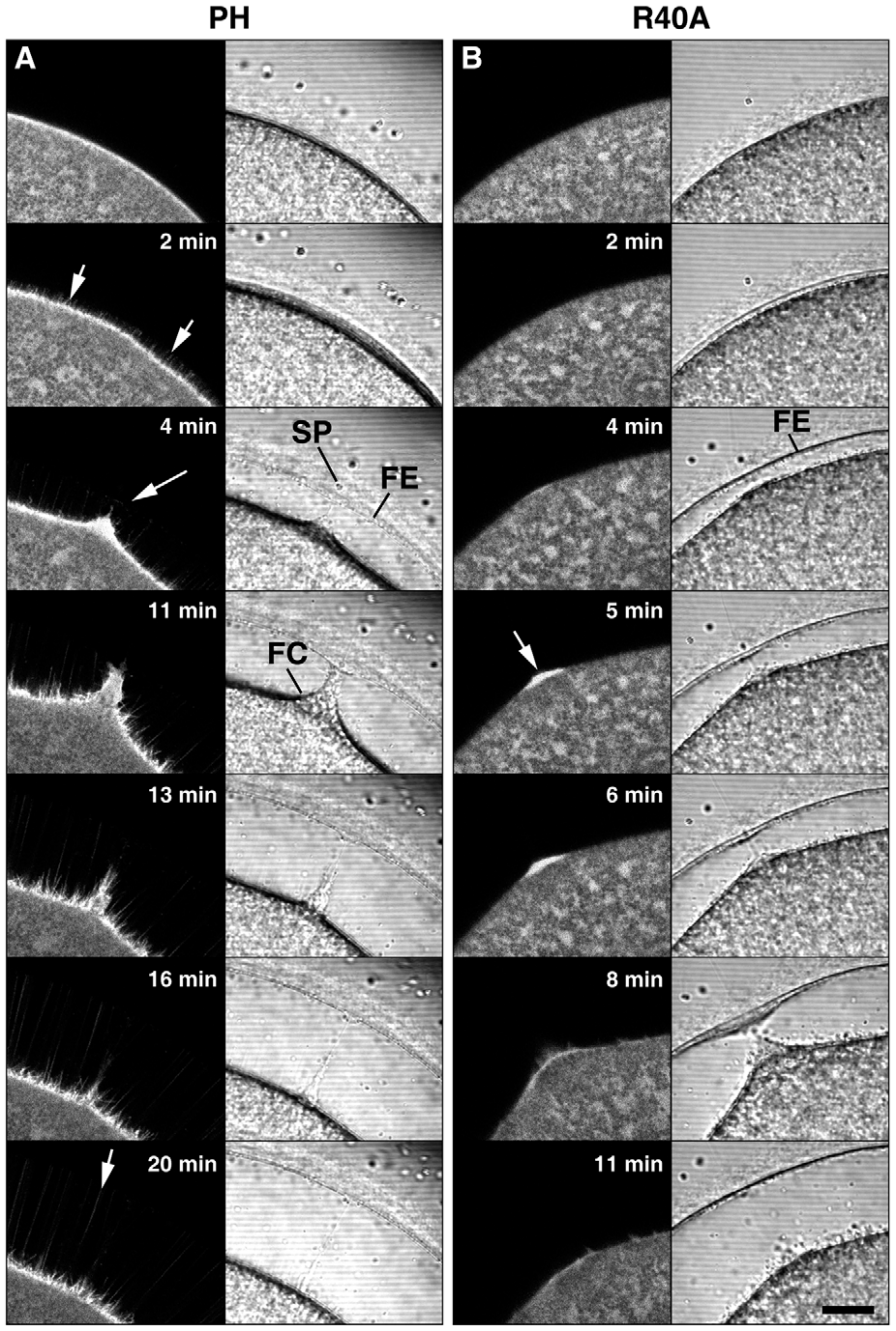
The increase of PIP2 at the fertilization site is accompanied by formation of numerous spikes that protrude the plasma membrane concomitantly with the fertilization envelope elevation. Mature eggs were microinjected either with RFP-PH (150 µM, pipette concentration) or with its control protein (R40A mutant) prior to fertilization. (A) Confocal microscopic images of RFP-PH fluorescence (left column) and the corresponding bright field views. The moment of sperm-egg contact was set to t = 0 (top panels). At 2 min, as the fertilization envelope started to be elevated, RFP-PH began to visualize spikes formation (white arrows). Those spikes perfectly laid on the confocal plane spanned the entire depth of the perivitelline space, and their length grew as the vitelline layer was elevated (arrow at 4 and 20 min). (B) The control probe R40A did not visualize spikes formation, but labeled the actin-rich fertilization cone (arrow at 5 min). Abbreviation: SP, sperm; FE, fertilization envelope; FC, fertilization cone.

**Figure 4:**
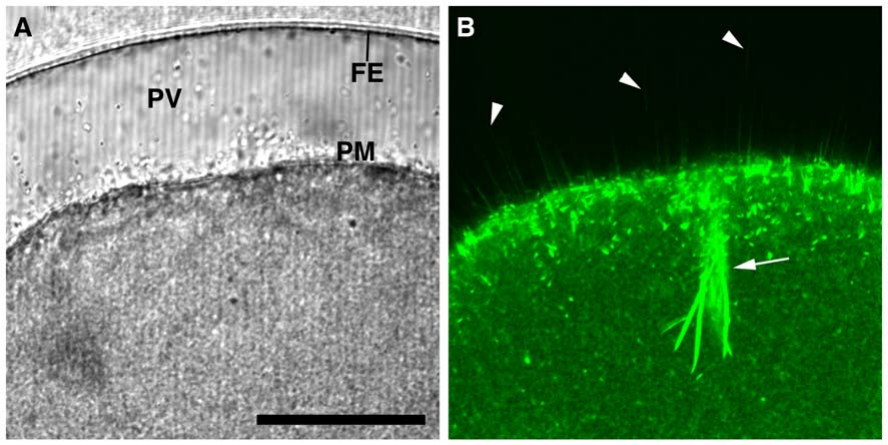
The spikes protruding into the perivitelline space during fertilization are composed of actin filaments. Mature eggs of *A. aranciacus* were microinjected with Alexa Fluor 488-phalloidin (50 µM, pipette concentration) prior to fertilization. (**A**) Transmission photomicrograph of the fertilized egg 10 min after insemination. (**B**) The corresponding confocal image of Alexa Fluor 488 phalloidin-stained F-actin. In addition to the strong staining of the actin bundles associated with the penetrating sperm (arrow), fluorescent phalloidin disclosed numerous spikes in the perivitelline space (arrowheads). Abbreviation: FE (fertilization envelope), PV (perivitelline space), PM (plasma membrane). Scale bar, 50 µm.

The physiological significance of the slow-rising second phase of the PIP2 increase at the plasma membrane (Fig. 2C) was studied further by confocal microscopy. By 2 min after sperm-egg contact, when the vitelline layer was visibly elevated to form the fertilization envelope, labeling of PIP2 by RFP-PH exhibited striking morphological changes at the egg plasma membrane (Fig. 3A). The intensity of RFP-PH signals at the plasma membrane was substantially increased (10.4% higher) in gray scale compared with the level at 0:00, suggesting that the second phase of RFP-PH increase at the plasma membrane was temporally correlated with the elevation process of the vitelline layer. Surprisingly, the labeling of membrane PIP2 with RFP-PH clearly delineated numerous spike-like structures (approximately 0.5 µm in diameter) protruding from the plasma membrane (Fig. 3A, arrows at 2 and 4 min). Concurrent with the elevation of the fertilization envelope, the spikes elongated and traversed the entire depth of perivitelline space (up to 30–50 µm), with their tips reaching the elevating fertilization envelope (arrows at 2, 4, and 20 min). To test if the spikes were filled with microfilaments, F-actin was stained with fluorescent phalloidin (Fig. 4). Phalloidin has been known to stabilize actin polymers and thereby interfere with some actin-based biological processes such as cell locomotion and chromatin ‘fetching’ during meiotic maturation [40], [41]. Since our goal is to visualize the spikes to demonstrate that they are made of F-actin, starfish eggs were microinjected with low doses of fluorescent phalloidin that do not have any inhibitory effect on the fertilization process of starfish eggs [14]. As expected, starfish eggs preinjected with Alexa Fluor 488- phalloidin (50 µM, pipette concentration) were able to undergo normal fertilization process and displayed numerous microfilament-filled spikes in the perivitelline space (Fig. 4B, arrowheads), as well as the thick actin bundles that were formed at the head of the penetrating sperm (Fig. 4B, arrow). Taken together, these observations suggest that the PIP2 increase at the plasma membrane of the fertilized egg is closely linked to the formation of microfilament-filled spikes in the perivitelline space.

Being labeled by RFP-PH but not by the R40A mutant probe, the spikes in the perivitelline space are believed to be a structure ensheathed with the PIP2-containing plasma membrane (Fig. 3). To our surprise, however, not only RFP-PH but also the mutant R40A probe heavily decorated the fertilization cone (Fig. 3B, arrow at 5 min), where a large amount of newly formed F-actin is concentrated [14], [42]. Since the probes with RFP alone uniformly diffused to cytoplasm without showing any preference to the fertilization cone (Data S1, arrow), these results raised an intriguing possibility that the PH domain of PLC-δ1 might have a physical interaction with newly formed F-actin under certain circumstances, regardless of its PIP2-binding configuration.

### Sequestration of PIP2 by RFP-PH causes structural changes in the subplasmalemmal actin network

**Figure 5:**
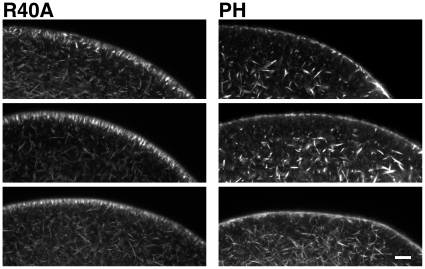
Sequestration of PIP2 by RFP-PH causes structural changes in the subplasmalemmal actin network. Mature eggs of *A. aranciacus* were microinjected with either RFP-PH or the R40A mutant proteins (330 µM, pipette concentration) and incubated for 25 min. The actin cytoskeleton was visualized by Alexa Fluor 488-conjugated phalloidin in three representative eggs for each treatment. Scale bar, 20 µm.

As PIP2 binds to an array of actin-binding proteins in cells [27], [28], [43], the fine structure of the actin cytoskeleton can be modified by altering the local levels of PIP2 [44]. Indeed, sequestering the plasma membrane PIP2 by over-expressing PH-GFP led to substantial reduction of the actin filaments in mammalian cells [38]. In line with these observations, microinjection of starfish eggs with RFP-PH (330 µM, pipette concentration) led to subtle changes in the organization of the actin cytoskeleton underneath the plasma membrane, as judged by Alexa Fluor 488-phalloidin. Whereas R40A-microinjected eggs displayed clustered actin bundles nearly perpendicular to the plasma membrane, as normally observed in mature eggs, the orderly organization of the actin filaments in the subplasmalemmal region was largely diminished in the eggs microinjected with RFP-PH (Fig. 5). Instead, the actin bundles resembling ‘pine spines’ were concomitantly enhanced in the inner cytoplasm.

### Sequestration of PIP2 by RFP-PH significantly delays the kinetics of the intracellular Ca^2+^ release

**Figure 6:**
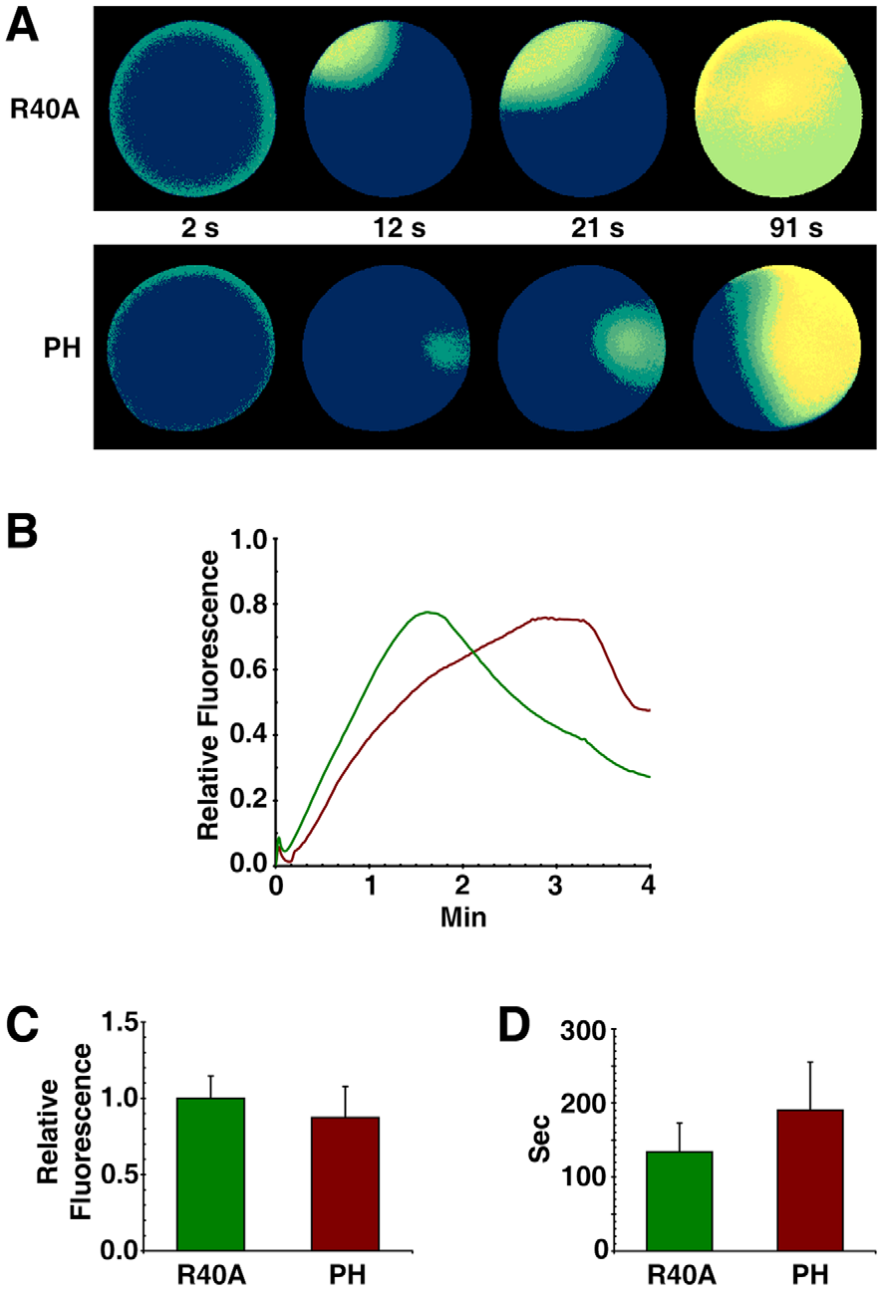
Sequestration of PIP2 by RFP-PH significantly delays the Ca^2+^ response at fertilization. *A. aranciacus* eggs preloaded with Ca^2+^ dye were microinjected with either RFP-PH or the R40A proteins (330 µM, pipette concentration). After 20 min incubation, the eggs were fertilized in the same experimental chamber (t = 0). (**A**) Visualization of the Ca^2+^ response in the paired eggs preinjected with RFP-PH or R40A. Relative fluorescence of Ca^2+^ signals was presented in pseudo-color images. Ca^2+^ rises in the eggs injected with R40A and RFP-PH were represented in green and brown curves, respectively. (**B**) Ca^2+^ signals quantified over the entire cytoplasmic field. (**C**) The magnitude of the Ca^2+^ response in the fertilized eggs preinjected with RFP-PH (0.873±0.205 RFU, n = 18) was slightly lower than that of the R40A-injected eggs (1.0±0.147, n = 19) (*P*<0.05). (**D**) The time required for reaching the peak of the Ca^2+^ release is much longer in the eggs injected with RFP-PH (190.4±85.0 sec, n = 18) than in R40A-injected eggs (134±3 8.7 sec, n = 19) (*P*<0.01). (All presented in Mean ± SD.).

**Figure 7:**
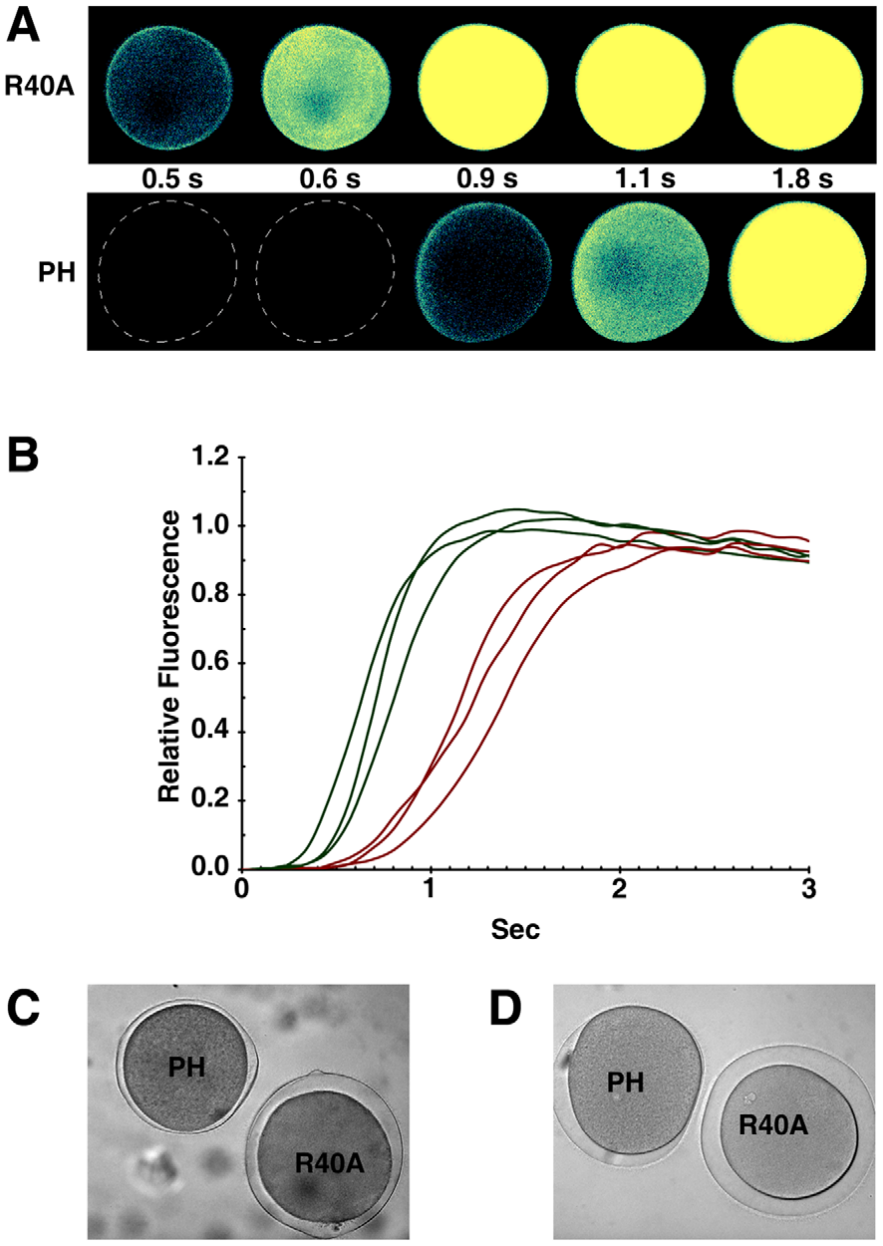
Sequestration of PIP2 by RFP-PH significantly delays the InsP_3_-induced Ca^2+^ release. *A. aranciacus* oocytes microinjected with Ca^2+^ dye and caged InsP_3_ were exposed to 1-MA for 1 h. Mature eggs were microinjected with either RFP-PH or the R40A mutant proteins (330 µM, pipette concentration), and were incubated for 20 min. (**A**) After photoactivation of the caged InsP_3_ (t = 0), the changes of cytosolic free Ca^2+^ were monitored by the relative fluorescence of Ca^2+^ signals. (**B**) Ca^2+^ signals quantified over the entire cytoplasmic field of the oocytes injected with RFP-PH and the R40A mutant proteins were represented in brown and green curves, respectively. (**C and D**) Bright field views of the eggs 12 min after InsP_3_ uncaging. Elevation of the vitelline layer after Ca^2+^ signaling is severely affected by RFP-PH.

PIP2 is coupled with InsP_3_-dependent Ca^2+^ signaling in several ways. As the substrate for PLC to produce InsP_3_, PIP2 is subject to hydrolysis during fertilization [45]. On the other hand, Ca^2+^-releasing activities of InsP_3_ receptors are increased several fold by anti-PIP2 antibodies in the reconstituted lipid bilayer, implying that PIP2 may rather repress InsP_3_-dependent Ca^2+^ signaling [46]. Furthermore, the InsP_3_-sensitive Ca^2+^-releasing mechanism can be modulated by the actin cytoskeleton [18], [19], suggesting that cytoskeletal changes induced by PIP2 may also indirectly influence Ca^2+^ signaling. To shed light on these seemingly contradictory roles of PIP2, we have sequestered plasma membrane PIP2 with RFP-PH and examined its effects on Ca^2+^ responses. As shown in Fig. 6, the magnitude of the Ca^2+^ response in the fertilized eggs preinjected with RFP-PH (Mean ± SD  = 0.873±0.205 RFU, n = 18) was slightly lower than that of the R40A-injected eggs (1.0±0.147, n = 19) (*P*<0.05), which was expected from the role of PIP2 as the substrate of PLC. When Ca^2+^ was released by photoactivation of the caged InsP_3_, sequestration of PIP2 at the plasma membrane with RFP-PH failed to influence the magnitude of the Ca^2+^ response, as the values for the eggs microinjected with RFP-PH (Mean ± SD  = 0.969±0.247 RFU, n = 15) and R40A (0.931±0.214 RFU, n = 15) were virtually the same (Fig. 7). The major effect of RFP-PH on Ca^2+^ signaling was rather on the kinetics. At fertilization, the time required for reaching the peak of Ca^2+^ release was much longer in the eggs microinjected with RFP-PH (Mean ± SD  = 190.4±85.0 sec, n = 18) than in R40A-microinjected eggs (134±38.7 sec, n = 19) (*P*<0.01) (Fig. 6D). Similarly, the photoactivation of caged InsP_3_ produced a much slower Ca^2+^ rise in the eggs microinjected with RFP-PH, as the Ca^2+^ peak was reached significantly later (Mean ± SD  = 1.77±1.03 sec, n = 15) than in the R40A-injected eggs (1.18±0.63 sec, n = 15) (*P*<0.05). To see if the delayed kinetics of the Ca^2+^ rise is due to the specific effect of RFP-PH on PIP2 binding, we have performed control experiments using neomycin that is known to bind PIP2 and inhibit PLC. It has been reported that rather a high concentration of neomycin is required to block Ca^2+^ signalling or PIP2 hydrolysis at fertilization, [29], [45]. However, by lowering the dose to 500 mM (concentration in injection pipette), we observed that the amplitude of the Ca^2+^ peak at fertilization was not significantly changed in neomycin-microinjected eggs (Mean ± SD  = 0.59±0.07 RFU, n = 7) in comparison with the control eggs (0.66±0.06 RFU, n = 5). On the other hand, the neomycin-injected eggs required significantly longer time (Mean ± SD  = 130.1±24.4 sec, n = 7) to reach the Ca^2+^ peaks in comparison to the control eggs (98.6±20.1 sec, n = 5) (*P*<0.05), which was in line with the results obtained with RFP-PH.

### Sequestration of PIP2 by RFP-PH interferes with vitelline layer elevation and leads to high frequency of polyspermy

As PIP2 sequestration had only modest effect on the magnitude of the Ca^2+^ response during fertilization and InsP_3_ uncaging (Fig. 6 and 7), it was expected that exocytosis of cortical granules and the resultant elevation of the vitelline layer would be normal in those eggs microinjected with RFP-PH. However, it was not the case. The elevation of the vitelline layer at the end of fertilization or InsP_3_ uncaging was often substantially impaired in these eggs. Most notably, the eggs microinjected with RFP-PH tended to display higher (more than two-fold) frequency of hemispheric impairment of vitelline layer elevation (see the egg labeled ‘PH’ in Fig. 7D), whereas the eggs microinjected with R40A behaved like the buffer-injected control eggs (Table 1). Even when the vitelline layer was elevated over the entire surface, the extent of its elevation in the eggs microinjected with RFP-PH was markedly compromised in comparison with the R40A-microinjected eggs (Fig. 7C). These results indicate that proper progression of cortical granule exocytosis may require additional factors other than Ca^2+^ signalling. One of such factors might be related to the structural changes of the actin networks induced by RFP-PH (Fig. 5), as similar inhibitory effects were observed when starfish eggs were treated with other agents affecting the cortical actin cytoskeleton, e.g. jasplakinolide and latrunculin-A [13], [14].

**Table 1:** Effects of RFP-PH on the formation of the fertilization envelope.

Ca^2+^-inducer	Effector		VL Elevation	
		Full	Partial	None
**InsP_3_**	Buffer (n = 11)	82%	18%	0%
	R40A (n = 17)	82%	12%	6%
	RFP-PH (n = 26)	46%	38.5%	5.5%
**Sperm**	Buffer (n = 87)	76%	23%	1%
	R40A (n = 20)	75%	20%	5%
	RFP-PH (n = 22)	40.9%	45.5%	13.6%

Mature eggs of *A. aranciacus* microinjected with either RFP-PH or R40A mutant proteins (330 µM, pipette concentration) were stimulated for Ca^2+^ signaling and cortical granule exocytosis by fertilization or photoactivation of uncaged InsP_3_. Partial elevation refers to the eggs displaying hemispheric impairment of fertilization envelope formation (Fig. 7D). Data were pooled from thee different batches of eggs at different breeding seasons. Abbreviation: VL, vitelline layer.

**Figure 8:**
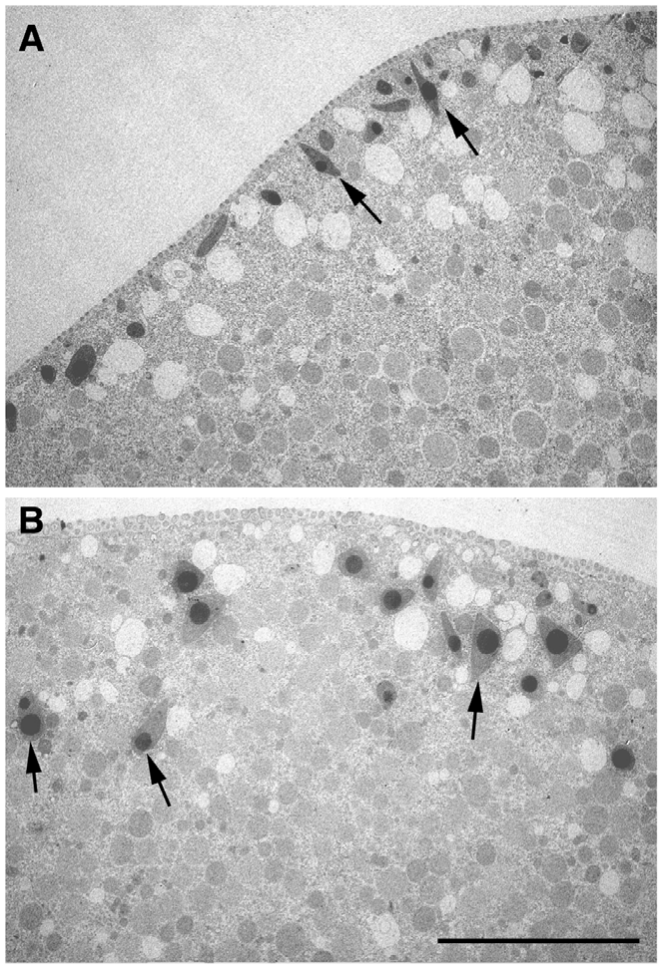
Sequestration of PIP2 by RFP-PH affects translocation of cortical granules during meiotic maturation. Transmission EM exhibited the ultrastructure of the eggs matured in the presence of RFP-PH or the R40A proteins (330 µM, pipette concentration). (**A**) In the eggs microinjected with R40A, cortical granules are intimately apposed to the plasma membrane, often perpendicular to the cell surface (arrows). (**B**) In the eggs microinjected with RFP-PH at the GV stage, cortical granules failed to be stacked underneath the plasma membrane, and were often located at a considerable distance from the plasma membrane (arrows). Scale bar, 10 µm.

Actin plays an essential role in translocation of the cortical granules to the plasma membrane during meiotic maturation [47], [48], and the sequestration of PIP2 by RFP-PH interfered with the intracellular transport, as judged by the location of cortical granules in the transmission electron microscopy image (Fig. 8, arrows). Moreover, in line with the deregulated actin cytoskeleton and the impaired cortical granule exocytosis, the fertilized eggs preinjected with RFP-PH displayed significantly high frequency of polyspermic fertilization with respect to the eggs preinjected with R40A (Table 2).

**Table 2:** Effects of RFP-PH on sperm-egg interaction.

Effector	No. fertilizing sperm
Buffer	1.20±0.45 (n = 5)
R40A	1.47±0.77 (n = 19)
RFP-PH	2.70±1.47 (n = 24)

Mature eggs of *A. aranciacus* loaded with Ca^2+^ dye were microinjected with either RFP-PH or R40A mutant proteins (330 µM, pipette concentration) and fertilized. The number of fertilizing sperm (Mean ± SD) refers to the number of initial spots of Ca^2+^ transient immediately after insemination. The average number of fertilizing sperm in the eggs preinjected with RFP-PH was significantly higher than those of the R40A-injected (*P*<0.01) and buffer-injected (*P*<0.001) control eggs, while the eggs injected with the buffer or R40A showed no significant difference from each other.

### Spike formation in the perivitelline space takes place not only in starfish eggs but also in sea urchin eggs at fertilization

**Figure 9:**
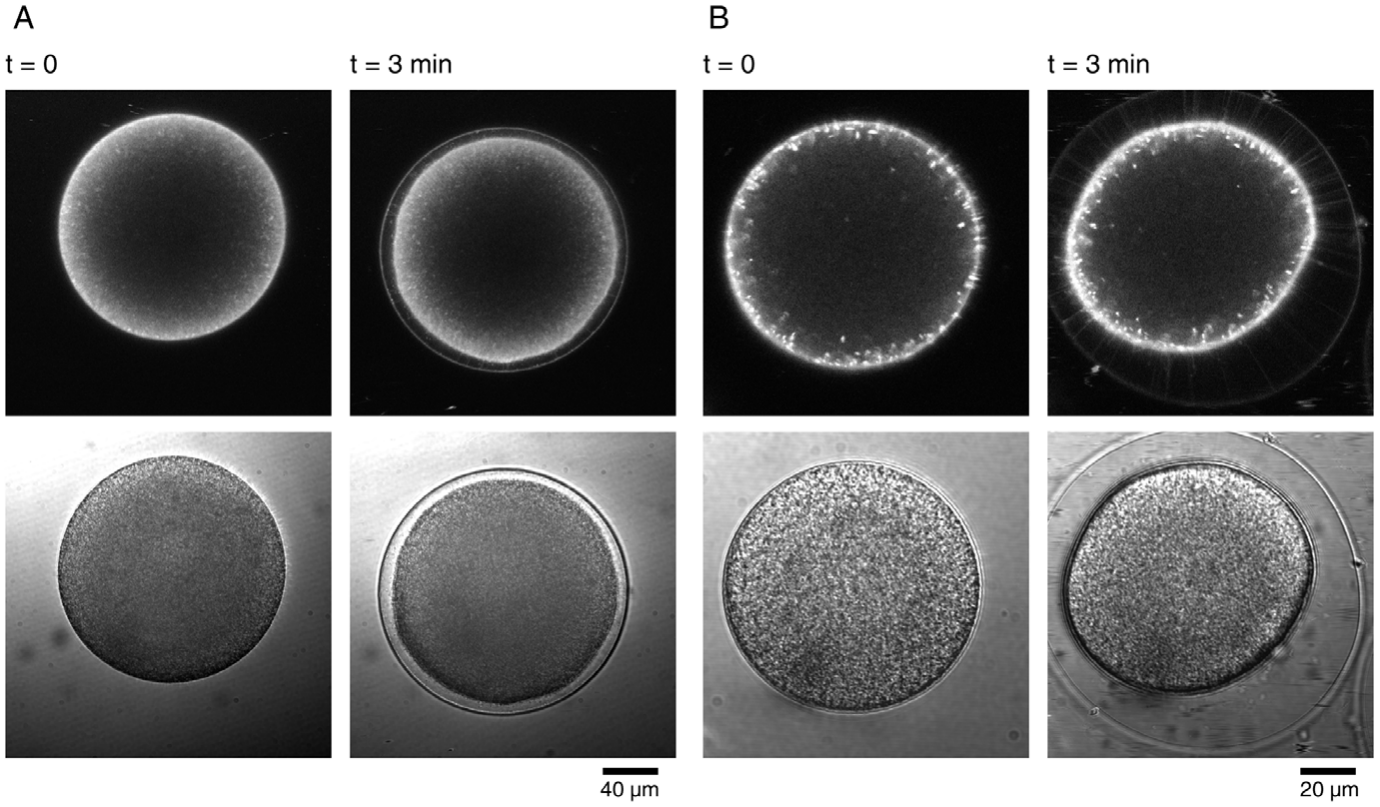
Perivitelline spike formation is not limited to starfish eggs, but is also present in sea urchin eggs at fertilization. Starfish (*A. pectinifera*) (A) and sea urchin (*P. lividus*) eggs (B) were stained with FM 1-43 as described in Materials and Methods. After immediate rinse with FSW, sperm were added (t = 0), and the eggs were imaged with confocal microscopy to monitor the spike formation and the elevation of the fertilization envelope.

To test if the spike formation in the perivitelline space is limited to a certain species of starfish or to a detection method (RFP-PH) (Fig. 3A), we visualized the perivitelline spikes in an alternative method. To this end, eggs of a different starfish species (*Asterina pectinifera*) and sea urchin (*Paracentrotus lividus*) were briefly stained prior to fertilization, by use of a lipophilic dye that selectively delineates the plasma membrane. As shown in Fig. 9, the egg activation after fertilization was much faster in sea urchin eggs. By 3 min after insemination, the fertilization envelope was fully elevated, and a myriad of spikes were visualized by FM 1-43 in the perivitelline space (Fig. 9B). In the same time scale, the starfish eggs were activated more slowly but displayed a similar pattern of spike formation in the perivitelline space (Fig. 9A).

## Discussion

PIP2 is a key molecule for cell signal transduction as it is the precursor of two second messengers, InsP_3_ and diacylglycerol. Furthermore, PIP2 by itself has other roles in regulating cytoskeletal structures and ion channel activities [26], [27]. As Ca^2+^ mobilization and dynamic restructuration of the actin cytoskeleton are the two major initial events at fertilization, it was of great interest to see how the local level of PIP2 changes at the site of fertilization. However, the issue of PIP2 metabolism at fertilization has been somewhat controversial. Upon fertilization, mouse eggs displayed an increase of PIP2 at the plasma membrane [31], but an apparent decrease of PIP2 was subsequently reported in sea urchin eggs [29]. Although these contradictory results may reflect species difference, earlier biochemical studies with sea urchin eggs also reported a significant increase of PIP2 at fertilization [30]. We noted that an experimental parameter that had potentially complicated the analyses might be the use of denuded eggs. In the two studies that produced opposite results with respect to the changes in plasma membrane PIP2 levels at fertilization [29], [31], the vitelline layer of sea urchin eggs and the zona pellucida of mammalian eggs were chemically removed by use of dithiothreitol (DTT) or Tyrode's acid. This procedure might have been inevitable to facilitate microinjection and imaging experiments in relatively small eggs. However, in the light of the fact that the reducing power of DTT and the drastic pH conditions could alter the activities of many other enzymes and proteins on the surface of the living eggs, the method is hardly free from iatrogenic effects. Here, we have used intact starfish eggs, which are large (about 300 µm in diameter) and transparent, without any artificial treatment to remove vitelline layers, and found that PIP2 actually had a multiphasic response at the plasma membrane during fertilization. Its brief initial change and the more prolonged late increase appear to have distinct physiological meanings. The level of PIP2 at the plasma membrane began to increase about 10 sec after the onset of Ca^2+^ signaling (Fig. 2B), but subsequently decreased amid a sharp rise of intracellular Ca^2+^ (Fig. 2C). This result could be attributed to the hydrolysis of PIP2 by PLC, which is activated by the propagating Ca^2+^ wave. Hence, the initial fluctuation of plasma membrane PIP2 level appears to be related to the production of InsP_3_. However, it bears an emphasis that the very initial Ca^2+^ signal took place without disturbing the plasma membrane PIP2 levels at all (Fig. 2C). This might have happened if PIP2 hydrolysis and synthesis had been in perfect equilibrium at the very onset of Ca^2+^ signaling. Alternatively, such conspicuous lack of a detectable decrease in PIP2 is in line with the idea that the initial trigger of the intracellular Ca^2+^ release at the fertilization of echinoderm eggs might be a second messenger different from InsP_3_, e.g. NAADP [3], [49]. Indeed, the increase of InsP_3_ occurred only *after* the major release of intracellular Ca^2+^ had taken place in the fertilized eggs of sear urchin, as judged by the decline of the plasma membrane PIP2 levels [29]. The massive increase of InsP_3_ well after the major release of intracellular Ca^2+^ at fertilization [29], [32] raises an intriguing question on the physiological significance of PIP2 hydrolysis besides Ca^2+^ signaling.

Our data showed that the late and prolonged increase of PIP2 was spatiotemporally linked to the elevation of the fertilization envelope (Fig. 2 and 3). The time-lapse confocal microscopic images clearly linked the PIP2 increase to the ongoing formation of spikes that was taking place concomitantly with the elevation of the fertilization envelope (Fig. 3C and Data S2). The rapid formation of numerous spikes was limited neither to the sperm-egg interaction site nor to the eggs of starfish, as it was also observed at the entire surface of sea urchin eggs at fertilization (Fig. 9). Hence, the formation of perivitelline actin spikes at fertilization may be a general phenomenon in echinoderm eggs, and their roles at the later stages of embryonic development would be an interesting topic of future investigations.

By nature, these spikes are reminiscent of microvilli, a myriad of small finger-like protrusions of the plasma membrane which are involved in various functions including absorption and mechanotransduction. Microvilli on the egg surface also play a role in gamete fusion [50]. In immature oocytes of starfish, the small microvilli (0.15 µm thick and 0.4 µm high) have a potential to thicken and elongate following hormonal stimulation [51]. In this communication, we report that microvilli have even more ‘elastic’ nature in a living cell. When microinjected with the PIP2-binding probes (PH-GFP or RFP-PH), the live oocytes of starfish have exhibited highly dynamic microvilli, continually fluctuating on the cell surface (Data S3). Like microvilli, which are filled with actin filaments that are at dynamic equilibrium, the spikes formed in the perivitelline space of eggs following fertilization contained microfilaments, as demonstrated by fluorescent phalloidin (Fig. 4). However, they were remarkably longer (up to 50 µm) and thicker (0.5 to 1.0 µm) than microvilli (Fig. 3). According to the freeze-fracture studies on sea urchin eggs, microvilli quickly undergo structural changes upon fertilization, with their diameter more than doubled within 2 min [52]. Since both spikes and microvilli contain PIP2 and microfilaments, it is conceivable that the spikes may originate from the microvilli that are thickened and elongated at fertilization [53]. Alternatively, the spikes might be sprouted *de novo* by the action of microfilaments and actin-binding proteins.

The increase of PIP2 and actin filaments in a tight space inside the spikes of fertilized eggs raises an interesting question about the causal relationship between the two events. The late increase of PIP2 at the plasma membrane of fertilized eggs might simply reflect the increased surface of lipid bilayer as a result of cortical granule exocytosis [31]. However, PIP2 is known to be relatively scarce outside the plasma membrane [37], while fertilization is accompanied by a two fold increase of the net content of PIP2 in the eggs [30], [54]. Thus, the increase of PIP2 at the egg plasma membrane is likely due to its new synthesis following fertilization. While the initial increase of PIP2 may be for sustaining InsP_3_-based Ca^2+^ signaling by boosting its level, the prolonged overcompensation of PIP2 after the propagation of massive Ca^2+^ wave implies an additional role. Modulation of PIP2 levels with PIP2-sequestering proteins or with metabolic enzymes had an impact on the actin cytoskeleton in various cells [38], [55], [56]. Considering that local PIP2 levels regulate the actin cytoskeleton [28] and that PIP2-sequestration changes in the subplasmalemmal actin cytoskeleton (Fig. 5), it is tempting to speculate that the purpose of the late PIP2 increase is to induce spike formation by extending microfilament-filled spikes in the perivitelline space (Fig. 3 and 4).

The PH domain of PLC-δ1 binds to PI(4,5)P2 with high specificity and affinity (Kd = 1–5 µM) [25]. However, PIP2 may also be occupied by abundant proteins, e.g. profilin, at the plasma membrane [57]–[59]. Nonetheless, over-expression of the PH domain of PLC-δ1 has been used as a successful method to mask biological functions of PIP2 in various cell types [38], [39]. Whereas the approaches of over-expression by cell transfection may have a difficulty in estimating the precise amount of proteins being delivered to the cell, microinjection of purified proteins can circumvent this problem. We have used bacterially expressed RFP-PH as a mean to inhibit PIP2 in order to study its function at fertilization. In starfish eggs, the binding of RFP-PH to the accessible PIP2 was saturated at 330 to 660 µM (concentration in the injection pipette), and the lowest limit of the saturating dose (330 µM) was used for our analyses.

We have demonstrated that sequestration of PIP2 with RFP-PH disrupted the orderly organization of the subplasmalemmal actin network (Fig. 5). Alteration of the actin cytoskeleton with PH-GFP significantly reduces its adhesion to the plasma membrane in NIH-3T3 cells [38]. Subtle it may seem, the structural and physical changes caused by RFP-PH in starfish eggs were linked to higher incident of partial hemispherical elevation of the vitelline layer at the end of fertilization or InsP_3_ uncaging (Table 1), suggesting a role for PIP2 in the concerted elevation of the fertilization envelope. Interestingly, PIP2 displayed spherically asymmetrical distribution at the plasma membrane (Fig. 1D), as was reported in the mouse eggs [31]. Furthermore, sequestration of PIP2 with RFP-PH led to increased tendency of polyspermic fertilization (Table 2), which was typically observed at the regions where subplasmalemmal actin network was altered [14]. As successful formation of the fertilization envelope requires precise control of actin assembly and disassembly at the egg cortex [13], [14], the impaired elevation of fertilization envelope and the increased tendency of polyspermic interaction by sequestration of PIP2 might well be due to the alteration of the actin cytoskeleton.

With the dose at which its binding to plasma membrane PIP2 is nearly saturated (330 µM in injection pipette, i.e. 3.3 µM in cytoplasm), we have observed that the RFP-PH protein inhibits the sperm-induced Ca^2+^ signals, but only modestly (Fig. 6C). It is conceivable that the plasma membrane PIP2 molecules are already masked by other proteins that may be readily removed by the fertilization signals to permit an access to PLC. Alternatively, it cannot be ruled out that extra-plasmalemmal PIP2, which is not sequestered by RFP-PH, might contribute to the formation of InsP_3_-dependent Ca^2+^ signaling. At any rate, the primary effect of RFP-PH on the intracellular release of Ca^2+^ at fertilization or in response to uncaged InsP_3_ was the delayed kinetics in its rise rather than the changes of the magnitude (Fig. 6 and 7). The precise cause of this effect is difficult to tell. As PIP2 regulates ion channels [26], [46], it is conceivable that its sequestration has interfered with this aspect of the ion channel activities. Alternatively, since the PH domain of the PLC-δ1 also binds to InsP_3_ [33], RFP-PH might have momentarily buffered the uncaged or synthesized InsP_3_ and consequently delayed the Ca^2+^ response. On the other hand, this conspicuous latency in Ca^2+^ response might reflect the structural changes in the egg cytoplasm, as the reorganization of the actin cytoskeleton in the microenvironment of the Ca^2+^ stores could be also responsible for the delay in Ca^2+^ signaling [60].

According to the prevailing view, the Ca^2+^-evoked exocytosis of cortical granules deposits their contents into the perivitelline space with the assistance of protease activities [61], [62], which sever the vitelline layer form the plasma membrane. The extracellular matrix then swells up by osmotic pressure and solidifies as a result of peroxidase-based cross-linking of proteins [63]. Our report of microfilament-filled spikes in the perivitelline space adds a new dimension to this model of fertilization envelope elevation. That is, actin polymerization progressing at 2.5 to 5 µm/min (Fig. 3 and 4) underneath the elevating vitelline layer may provide a directed mechanical force to thrust the egg plasma membrane, as was demonstrated in other cells [64], [65]. In support of the idea, fertilized starfish eggs pretreated with the actin-depolymerizing drug latrunculin-A failed to elevate fertilization membrane despite a massive Ca^2+^ signaling [14]. However, deciphering the precise physiological role of the spikes formed in the perivitelline space of the echinoderm eggs at fertilization requires further investigation. One technical difficulty is that the agents that change the actin spikes can also affect the polymerization status of the subplasmalemmal actin networks, which were demonstrated to play important roles in sperm interaction and in the elevation of the fertilization envelope [14].

As exocytosis results in membrane fusion, the surface area of the plasma membrane is expected to increase during cortical granule exocytosis. However, this could be also counterbalanced by the subsequent site-specific compensatory endocytosis that is induced by the increase of intracellular Ca^2+^ [66]. Indeed, the starfish eggs (*A. pectinifera*) at fertilization displayed no evident increase in lipophilic labeling at the baseline of the plasma membrane (Fig. 9A). Nonetheless, the presence of numerous actin spikes containing PIP2 in the perivitelline space inevitably suggests their participation in establishing the net balance between the two opposite membrane trafficking by accommodating the increased membrane surface at the time of cortical granule exocytosis. In parallel with the changes of the actin cytoskeleton in the fertilized egg, the acrosome reaction in starfish sperm involves an extensive polymerization of actin, which protrudes as a long process (up to 90 µm) within just 30 sec [67]. This microfilament-filled ‘acrosomal processes’ establish a contact with the egg cortical actin that appears to promote the engulfment of the sperm [14]. Hence, a strategy involving actin polymerization is employed for fertilization by both gametes of starfish.

## Materials and Methods

### Preparation of oocytes

The Mediterranean species of starfish (*Astropecten aranciacus*) were captured in the Gulf of Naples during the breeding season (January to June) and maintained in circulating cold seawater (16°C). The gonads containing oocytes were dissected from the central dorsal area near the arms and transferred to the filter-sterilized cold seawater. Free oocytes were isolated as single cells by sieving through gauze several times followed by repeated rinsing and low speed (<1,000 rpm) sedimentation in cold filtered seawater. For fertilization experiment, immature oocytes were stimulated with 10 µM of 1-methyladenine (1-MA) for 1 h before being exposed to the spermatozoa in the filtered seawater.

### Construction of the plasmid expressing PH-GFP and RFP-PH

The PH domain of rat PLC-δ1 (amino acids 1-140) was prepared from RNA of Wister rat brains by RT-PCR and ligated into pCRII-TOPO vector (Invitrogen). To facilitate ligation and to remain in frame, restriction site sequences and a few extra nucleotides were added to the primers whenever necessary. After DNA sequence verification, the cDNA were ligated to the pET-28b vector (Novagen), which was already ligated to the GFP cDNA. The histidine-tagged protein expressed from the resulting vector contained the PH domain at the N-terminus side of GFP, and was accordingly named PH-GFP. To make RFP-PH, the cDNA for RFP was amplified from pCDNA3.1-HSP-RFP (a generous gift from Dr. D. Lim, University of Padova) in a similar procedure, and ligated to the pET28b vector, which already contained the PH domain. Between the PH domain and fluorescent tag, we inserted a tether of 5 glycine residues by adding the coding sequences to the PCR primers. The intermediate plasmid constructed in this procedure expressed either the fusion protein or the PH domain alone. The plasmid producing R40A mutant version of the PH fusion proteins were prepared from RFP-PH and PH-GFP plasmid by use of the QuickChange site-directed mutagenesis kit (Strategen) and the targeting primer pairs: R40f (5′-gtggcgtagggaagccttctacaagctac-3′) and R40r (5′- gtagcttgtagaaggcttccctacgccac-3′).

### Bacterial expression and purification

*E.coli* strain BL12 was transformed with the plasmid through the chemical method (heat shock at 42°C for 2 min). After overnight growth on LB/kanamycin plates and recovery at 4°C for >6 h, green or red colonies were selected for mass production. The overnight inoculates containing the transformed *E. coli* were diluted 10 fold, and further grown in 250 ml LB/kanamycine. After 90 min incubation at 30°C, the transformed *E. coli* were stimulated with 1 mM IPTG at 20°C for 18 h. The bacterial pellet was resuspended and sonicated in the lysis buffer containing 50 mM Tris, pH 7.9, 300 mM NaCl, 10 mM imidazole, 1 mM PMSF and the protease inhibitors CØmplete (Roche). Supplemented with 1% Triton X-100 and agitated on ice for 15 min, the lysate was centrifuged 20,000× *g* at 4°C for 30 min to collect soluble fraction. The supernatant containing the fusion proteins was applied to nickel-affinity chromatography (GE Health Care), and the imidazole-eluted 6H fusion protein was dialyzed against 10 mM Hepes buffer, pH 7.0 at 4°C for 4 h and supplemented with 100 mM L-Asp (injection buffer). The dialyzed protein was concentrated in Micron columns (Millipore) and adjusted to the desired concentration. The final protein was typically >95% pure, as judged by the coomassie-stained SDS-PAGE analysis.

### Microinjection, caged compounds, confocal microscopy, and video imaging

Microinjection of the oocytes was performed with an air-pressure Transjector (Eppendorf). For calibration of the injected volume for each substance, a predefined volume of the sample was delivered into the egg by use of Narishige IM16 readout type microinjector (Narishige Co., LTD, Japan) and used for the standard. The amount of injected material was targeted to be 1% of oocyte volume, and the final concentration of the injected material was thus estimated 100-fold lower than the concentration in the injection pipette. In the case of RFP-PH, the intensity of the plasma membrane labeling was proportionally increased with the dose of the injected protein up to 330 µM (concentration in pipette). Beyond this concentration, the labeling in the plasma membrane was saturated, and the surplus probes were remained in the cytoplasm. The dose of RFP-PH used to delineate the plasma membrane (150 µM in pipette) was more than two-fold lower than the saturation point, and did not have an effect on Ca^2+^ signaling or other physiological traits of the eggs (data not shown). Fluorescent calcium dye (Calcium Green) conjugated with 10 kDa dextran (Molecular Probes) was used in 500 µM (pipette concentration). Caged InsP_3_ (Molecular Probes) and the bacterially expressed proteins were prepared in the injection buffer (10 mM Hepes, pH 7.0, 100 mM L-Asp). To activate the caged InsP_3_ (Molecular Probes), microinjected oocytes were irradiated with 330 nm UV light for 25 seconds by using the computer-controlled shutter system Lambda 10-2 (Sutter Instruments, Co., Novato, CA). Alexa Fluor 488-phalloidin (Molecular Probes) was injected as described previously [13]. The microinjection protocol of neomycin (Sigma-Aldrich) conformed to the method specified in earlier publication [45]. To immobilize the eggs during imaging, the individual eggs were placed into a tight space created by two fused pieces of cover slips in the imaging chamber. Cytosolic Ca^2+^ changes were detected using a cooled CCD camera (MicroMax, Princeton Instruments, Inc., Trenton, NJ) mounted on a Zeiss Axiovert 200 microscope with a Plan-Neofluar 20x/0.50 objective. The quantified Ca^2+^ signal at a given time point was normalized to the baseline fluorescence (F_0_) following the formula F_rel_  =  [F-F_0_]/F_0_, where F represents the average fluorescence level of the entire oocyte. The incremental changes of the Ca^2+^ rise was analyzed by applying the formula F*_inst_* = [(F_t_-F_t_-_1_)/F_t_-_1_] in order to visualize the site of instantaneous Ca^2+^ release. Likewise, the instantaneous changes of PIP2 were assessed by the same formula. PIP2 labeling in the plasma membrane was compared with that in cortical cytoplasm by applying the formula F_rel_ = [F_pm_-F_ct_]/F_ct_, where F_pm_ and F_ct_ refer to the levels of fluorescence in the plasma membrane and the cortical cytoplasm, respectively. Fluorescence of Ca^2+^ and PIP2 images were analyzed with the MetaMorph Imaging System software (Universal Imaging Corporation, West Chester, PA). In the simultaneous monitoring of the intracellular Ca^2+^ response and the changes in the egg morphology and PIP2 levels at the sperm entry site (Fig. 2), the time-resolution of the time-lapse microscopic analyses was on the average 4.53 sec. During that time interval, the images of RFP fluorescence (150 ms), fluorescent Ca^2+^ dye (50 ms), transmission photomicrograph (50 ms) were captured by the cooled CCD camera, and the data were transmitted to the computer for recording.

### Statistical analysis

The numerical MetaMorph data were compiled and analyzed with Excel of Microsoft Office 2003. The average and variation of the data were reported as ‘mean ± standard deviation (SD)’ in all cases in this manuscript. The paired t-test was performed by use of Prism 3.0 (GraphPad Software, La Jolla, CA, USA), and *P<0.05* was considered as statistically significant.

### Transmission electron microscopy

Immature oocytes at prophase I (*Astropecten aranciacus*) were microinjected with of RFP-PH or its R40A (330 µM pipette concentration) and stimulated with 1-MA (10 µM) incubation for 1 h. The resulting oocytes at metaphase I (before the extrusion of the first polar body) were fixed in filtered seawater containing 1% glutaraldehyde (pH 8.0) for 1 h at room temperature and then treated with 1% osmium tetroxide for 1 h. Specimens were dehydrated in increasing concentrations of alcohol and embedded in EPON 812. Sections were stained with 2% uranyl acetate and 0.2% lead citrate and examined with a LEO 912 AB energy filter transmission electron microscope.

### Staining of fertilized eggs with the lipophilic dye

Starfish (*Asterina pectinifera*) and sea urchin (*Paracentrotus lividus*) eggs were treated with 1M citric acid for 30 sec and washed with FSW. To delineate the plasma membrane, the eggs were then stained by incubating with 4 or 1 µM of FM 1-43 (Molecular Probes), respectively, in FSW for 2 min. After rinse with FSW, sperm were added and the fertilized eggs were imaged with confocal microscopy for real-time monitoring.

## Supporting Information

Data S1 Fertilization of *A. aranciacus* eggs preinjected with RFP. Left panel: fluorescent view of the fertilized egg at the confocal plane transecting the fertilization cone. Right panel: the corresponding bright field view. Scale bar  = 20 µm.
(3.96 MB DOC)

Data S2 A movie clip of video animation showing the formation of fertilization envelope. The microfilament-filled spikes were visualized with RFP-PH, and superimposed with bright field view images to delineate the perivitelline space.
(3.01 MB AVI)

Data S3 A short movie clip of video animation showing the fluctuating microvilli on the surface of a starfish oocyte. Following microinjection with PH-GFP, a serial of 10 sequential images were taken by confocal microscopy (47 sec intervals) and composited in MetaMorph.
(1.88 MB AVI)
