# Supplementary figures and images for: The Biphasic Increase of PIP2 in the Fertilized Eggs of Starfish: New Roles in Actin Polymerization and Ca2+ Signaling
Source: PLoS One. 2010 Nov 23;5(11):e14100. doi: 10.1371/journal.pone.0014100 (PMC2990714; doi:10.1371/journal.pone.0014100)

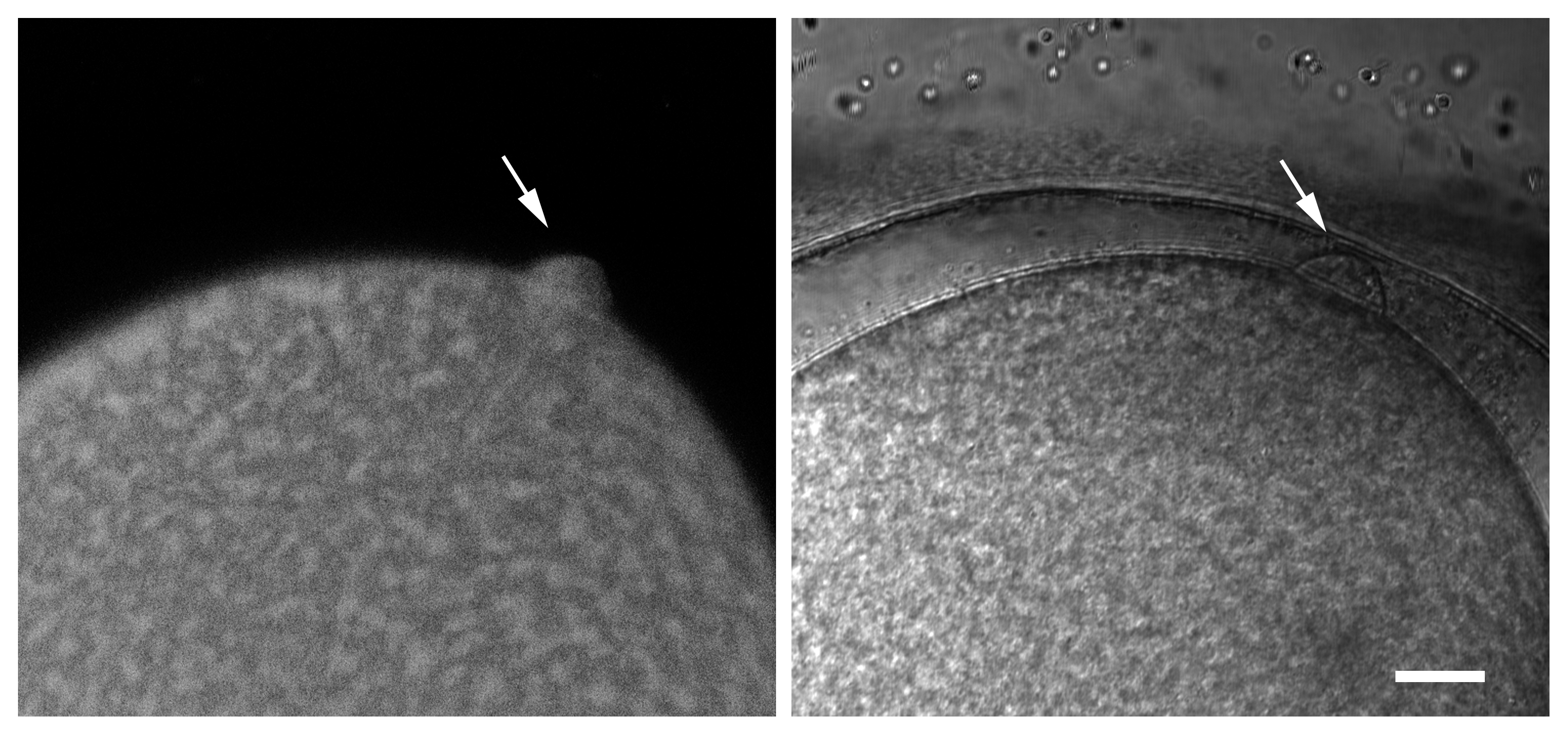

Supplement: Data S1 — Fertilization of A. aranciacus eggs preinjected with RFP. Left panel: fluorescent view of the fertilized egg at the confocal plane transecting the fertilization cone. Right panel: the corresponding bright field view. Scale bar = 20 µm. (3.96 MB DOC) [file pone.0014100.s001.tif]
